# Catalyst-Free Hydrolytic Polycondensation of Phenyltrimethoxysilane Under Ultrasonic Irradiation

**DOI:** 10.3390/polym18192347

**Published:** 2026-09-26

**Authors:** Nikita Yakhontov, Olga Gorbatsevich, Nina Demchenko, Georgy Cherkaev, Alina Khmelnitskaia, Aleksandra Kalinina, Aziz Muzafarov

**Affiliations:** 1 Enikolopov Institute of Synthetic Polymeric Materials, Russian Academy of Sciences, 117393 Moscow, Russia; yahontov@ispm.ru (N.Y.); gorbatsevich@ispm.ru (O.G.); alina.khmelnitskaya@ispm.ru (A.K.); aziz@ispm.ru (A.M.); 2 Lomonosov Institute of Fine Chemical Technologies, MIREA—Russian Technological University (RTU MIREA), Vernadskogo Avenue 78, 119454 Moscow, Russia; 3 Nesmeyanov Institute of Organoelement Compounds, Russian Academy of Sciences (INEOS RAS), Vavilova 28, 119991 Moscow, Russia

**Keywords:** catalyst-free hydrolytic polycondensation, ultrasonic irradiation, alkoxysilanes, phenyltrimethoxysilane, polyphenylsilsesquioxanes

## Abstract

Catalyst-free, solvent-free hydrolytic polycondensation of phenyltrimethoxysilane under ultrasonic irradiation was investigated at different reagent molar ratios. The effects of ultrasonic power, temperature, and irradiation time on monomer conversion, functional-group conversion, and product composition were evaluated. The optimal conditions for ultrasound-assisted, catalyst-free hydrolytic polycondensation of phenyltrimethoxysilane were determined. The products were characterized using a combination of physicochemical methods, including ^1^H and ^29^Si NMR spectroscopy, IR spectroscopy, elemental analysis, GPC, GC–MS, and TGA.

## 1. Introduction

Currently, one of the major challenges in polymer science is the development of environmentally sustainable routes to polymers. In line with the principles of green chemistry [1,2,3], current approaches aim not only to minimize the environmental impact of polymer production, but also to achieve precise control over polymerization processes in terms of the structure–property relationship of the product. In organosilicon chemistry, an example of this approach is the concept of “chlorine-free silicone chemistry” [4]. In particular, it involves the replacement of established chlorosilane-based processes with alkoxysilanes in hydrolytic polycondensation (HPC). Beyond reducing the environmental impact, this strategy improves control over the HPC process.

Recently developed approaches to the activation of catalyst-free HPC have demonstrated considerable potential when applied to methyltrialkoxysilanes. These approaches include ultrasonic irradiation [5], microwave irradiation [6], and increasing the vapor pressure of reaction mixture components [7]. They enable the preparation of previously inaccessible, highly functional oligo(hydroxy)methylsilsesquioxanes that are soluble in aqueous–alcoholic media and stable during storage, while providing broad control over residual functionality and molecular weight characteristics.

In contrast, the use of physical-field activation for the HPC of phenyltrialkoxysilanes, as well as detailed characterization of the resulting structures, has received considerably less attention in the literature. To date, such studies are essentially limited to the hydrolytic polycondensation of phenyltrimethoxysilane (PhTMS) in subcritical water [8]. Data on other systems are practically nonexistent. This prompted us to investigate the effect of these activation methods with the aim of obtaining analogous products with other substituents at the silicon atom, such as phenylsilsesquioxanes.

Polyphenylsilsesquioxanes are attractive functional materials, and synthetic approaches that enable precise control over their structure offer broad opportunities for practical applications. Products of partial hydrolysis of phenyltrichlorosilane, for example, have been used as additives for construction mortars [9,10], for the treatment of natural fibers in composite materials production [11,12], and in hydrophobic coatings [13,14]. Phenylsilsesquioxane and phenylsilsesquioxane–methylsilsesquioxane copolymer resins are also used as components of binders for fiberglass composites [10]. Heat-resistant enamels and varnishes, including KO-85, KO-815, and F-9 (Russian enamel brands), can be prepared by incorporating acrylic or glyphthalic resins into phenylsilsesquioxane-based systems [10].

Modern controlled-synthesis approaches give access to polyphenylsilsesquioxanes with tunable structural parameters and a variety of architectures—highly branched acyclic, star-shaped, nanogel [15,16], macrocyclic [16,17], polyhedral, and ladder-like [18]. Ladder-like materials are of particular interest for gas-separation membranes [19], photoresists [20], and composite materials [21]. However, the potential of catalyst-free approaches to obtain polyphenylsilsesquioxane via HPC remains unexplored.

Therefore, the aim of this work was to investigate the catalyst-free hydrolytic polycondensation of phenyltrimethoxysilane under ultrasonic irradiation and to determine how the process conditions affect monomer conversion, functional group conversion, and the composition and molecular weight characteristics of the products.

## 2. Materials and Methods

### 2.1. Materials

All reagents were commercially available: phenyltrimethoxysilane and trimethylchlorosilane (ABCR, Karlsruhe, Germany), methyl tert-butyl ether, toluene, THF, and hexamethyldisilazane (ECOS-1, Moscow, Russia). The reagents were purified using standard procedures [22]. Trimethylchlorosilane, hexamethyldisilazane, and THF were distilled at atmospheric pressure. Toluene was purified and dried by distillation over CaH_2_. Phenyltrimethoxysilane was distilled under vacuum immediately before use.

Ultrasonic treatment of the samples was performed using a system consisting of an Inlab I10–2.0 ultrasonic generator operating at 22 kHz with an adjustable power of 0–2000 W, equipped with an Inlab PMS–2.0/22 magnetostrictive transducer and an immersion waveguide probe.

### 2.2. Characterization Methods

Gas–liquid chromatography (GLC) analysis was performed on a Chromatek Analytic 5000 chromatograph (CHROMATEC, Yoshkar-Ola, Russia), with a katharometer detector, a helium carrier gas, 2 m × 3 mm columns, and a stationary phase SE-30 (5%), printed on Chromaton-H-AW. Registration and calculation of data were performed using the program “Chromatek Analyst” (CHROMATEC, Yoshkar-Ola, Russia).

Gel-permeation chromatography (GPC) was performed on a chromatographic system consisting of a STAIER series II high-pressure pump (Aquilon, Nakhodka, Russia), a RIDK 102 refractometric detector (Waters Corporation, Prague, Czech Republic), and a JETSTREAM 2 PLUS column thermostat (KNAUER, Berlin, Germany). The temperature was controlled at 40 °C (±0.1 °C). Tetrahydrofuran was used as the eluent; the flow rate was 1.0 mL/min. A 300 × 7.8 mm column filled with Phenogel sorbent (Phenomenex, Torrance, CA, USA), particle size 5 μm, pore size 10^3^ Å was used (passport separation range–up to 75,000 Da). Recording and processing of data were carried out using UniChrom 4.7 software (Minsk, Belarus).

The molecular masses of the products were determined relative to linear polystyrene standards. Typical GPC curves are provided in the Supplementary Materials, Figures S4–S6.

^1^H nuclear magnetic resonance (NMR) spectra were recorded at room temperature on a Bruker WP 250 SY spectrometer (250.13 MHz) for all samples. Chemical shifts (δ) in ppm were calibrated to residual solvent peaks ((CD_3_)_2_CO: = 2.05 ppm). The spectra were processed in ACD LABS software v.10.

^29^Si NMR spectra were recorded at room temperature on a Bruker Avance II 300 (59.64 MHz). Chromium (III) acetylacetonate was used as a relaxation agent.

IR spectra were recorded on an IR Fourier spectrometer—Nicolet iS50 (Thermo Scientific, Waltham, MA, USA)—in the ATR mode at a resolution of 4 cm^−1^ with 32 scans for each wave number in the range of 1000–4000 cm^−1^.

Gas chromatography–mass spectrometry (GC-MS) analysis of the initial compounds was performed on a Shimadzu GCMS-QP2020 Gas Chromatograph Mass Spectrometer with an SH-RTx-5MS column (30 m × 0.25 mm i.d. × 0.25 μm, Shimadzu, Kyoto, Japan), electron impact (EI) ionization method, and single quadrupole detector (positive ions).

Elemental analysis was performed on a Carlo Erba 1106 instrument (Milan, Italy). The relative error in determining silicon, carbon, and hydrogen contents did not exceed 0.1 wt%. Silicon, carbon, and hydrogen contents were determined by combustion of a sample (510 × 10^–3^ g) in an oxygen atmosphere at a temperature of 950 °C.

Thermogravimetric analysis (TGA) was performed at Moscow State University (Russia) using a TGA/DSC 3+ synchronous thermal analyzer (Mettler Toledo, Greifensee, Switzerland). The measurements were performed in air under heating from 30 to 600 °C at a rate of 10°/min.

### 2.3. General Method for Catalyst-Free HPC of PhTMS Under Ultrasonic Irradiation

PhTMS and water were added to a 50 mL reactor equipped with a water-cooling jacket. The probe was immersed in the reaction mixture to a depth of 5–8 mm; a schematic of the working section of the ultrasonic setup is given in the Supplementary Materials (Figure S1). Ultrasonic treatment is performed in continuous mode. The reagent loadings are listed in the Supplementary Materials (Table S1). The reactions were carried out at the temperatures, ultrasonic powers, and irradiation times specified in Table 1. Temperature was controlled using a continuous-flow water-cooling system. The reaction mixture yield was quantitative under all conditions studied. After irradiation, toluene was added to the reaction mixture, and the organic layer was separated from the aqueous–alcoholic layer and dried over Na_2_SO_4_. The organic products were then blocked with a mixture of trimethylchlorosilane (TMCS) and hexamethyldisilazane (HMDS).

The composition of the structural units in the product was determined by ^29^Si NMR with the addition of chromium (III) acetylacetonate.

^29^Si NMR Spectrum of the Product of the Hydrolytic Polycondensation of PhTMS: (((CD_3_)_2_CO), δ, ppm): −61.00 … −63.00 ppm (br., PhSiO_0.5_(OH)_2_); −68.50 … −70.00 ppm (br., PhSiO(OH)); −70.00 … −71.60 ppm (br., PhSiO(OMe)); −75.50 … −81.50 ppm (br., O_1.5_SiPh).

### 2.4. Catalyst-Free HPC of PhTMS Under Mechanical Stirring (Control)

15.86 g (0.08 mol) of PhTMS and 8.64 g (0.48 mol) of water (PhTMS/water = 1/6) were added to a 50 mL single-neck flask and stirred at 500 rpm at 35 °C for 3 days. PhTMS conversion was monitored by GLC.

### 2.5. Blocking of the Products

The toluene solution of the hydrolysis products (15 wt%) was added dropwise to a solution of TMCS and HMDS in toluene (30 wt%). The amounts of TMCS and HMDS were taken at a ratio of 1.2 mol per 1 mol of OH groups, based on their theoretical content of 40% in the resulting hydrolysis product. The reaction mixture was refluxed with stirring for 8 h, then washed with distilled water until the pH of the aqueous extract was neutral, dried over Na_2_SO_4_ for 12 h, and filtered. Toluene was removed using a rotary evaporator, and the residue was dried under vacuum at 110 °C/1 mmHg. Volatile products were analyzed by GLC and GC–MS. The residue was analyzed by ^1^H NMR, IR, and GPC. Based on the ^1^H NMR data, the empirical formula of the structural unit was calculated from the ratios of the integrated intensities of the PhSiO, MeOSi, and Me_3_SiO groups. The molecular masses of the products were determined by GPC relative to linear polystyrene standards. The extent of the process was confirmed by IR spectroscopy by the absence of absorption bands at 3600–3500 cm^−1^, characteristic of O–H stretching vibrations.

The reaction conditions and characteristics of the samples obtained are summarized in Table 1.

^1^H NMR spectrum of the typical blocked oligophenylsilsesquioxane product: (((CD_3_)_2_CO), δ, ppm): 0.03–0.21 (m, SiMe); 3.48–3.53 (m, OMe); 7.10–7.80 (m, SiPh).

FT-IR spectrum of the typical blocked oligophenylsilsesquioxane product (ATR mode): ν/cm^−1^: 3050 (C-H) weak; 2950 very weak; 2900 very weak; 2850 very weak; 1580 (C=C) weak; 1400 medium; 1076 (SiO) strong; 1020 (SiOSi) strong; 850 medium; 800 medium; 700 medium; 650 (SiC) medium; 550 (Si-C_6_H_5_) medium.

GC-MS spectrum of the low molecular weight fraction of the blocked PhTMS product (catalyst-free HPC): 241.0 [Si_2_C_10_H_17_O_3_]^+^; 299.0 [Si_3_C_12_H_23_O_3_]^+^; 357.1 [Si_4_C_14_H_29_O_3_]^+^; 451.1 [Si_4_C_19_H_31_O_5_]^+^.

Elemental analysis of sample № 18 (unblocked sample 8 in 2 years at RT): Found (%): Si, 20.94; C, 54.15; H, 4.69. Calculated (%): Si, 21.74; C, 55.78; H, 3.90.

Elemental analysis of sample № 19 (unblocked sample 8 in 5 years at RT): Found (%): Si, 20.99; C, 53.86; H, 4.72. Calculated (%): Si, 21.74; C, 55.78; H, 3.90.

Elemental analysis of sample № 20 (sample 2 after curing at 200 °C): Found (%): Si, 20.17; C, 56.24; H, 4.29. Calculated (%): Si, 21.74; C, 55.78; H, 3.90.

## 3. Results and Discussion

HPC of PhTMS under ultrasonic irradiation was investigated in the absence of both catalyst and solvent, using an ultrasonic reactor and an approach previously developed for methyltrimethoxysilane (MTMS) [5]. The ultrasonic power was varied from 300 to 1200 W, the reaction temperature from 15 to 55 °C, and the PhTMS/H_2_O molar ratio from 1/3 to 1/9. Product composition and structure were evaluated using the methodology described previously [5].

To analyze the composition of the PhTMS HPC products, the terminal Si–OH groups were blocked with trimethylchlorosilane under conditions that did not alter the oligomeric structure.

The efficiency of blocking was evaluated by IR spectroscopy (Figure 1), based on the disappearance of the absorption band at 3600–3500 cm^−1^ characteristic of O–H stretching vibrations.

The blocked products were distilled at 110 °C and 1 mmHg. The composition and structure of the volatile low-molecular weight fraction were analyzed by GLC and GC–MS, while the distillation residues, i.e., oligomeric products, were analyzed by GPC, IR, and ^1^H NMR spectroscopy.

By correlating the GLC and GC–MS data (Figure 2), with peak assignment to specific condensation products performed by GC–MS, the volatile fraction was shown to be a complex mixture of partial-hydrolysis products of PhTMS and their dimers. It also contained residual PhTMS, hexamethyldisiloxane formed during blocking of hydroxyl groups, and toluene.

The residual methoxy and hydroxyl contents were determined by ^1^H NMR from the integrated-intensity ratios of the PhSiO_1.5_, MeOSi, and Me_3_SiO signals, respectively. (Figure 3).

^29^Si NMR analysis of the as-synthesized PhTMS HPC product was used to determine its general structural formula by comparison of the observed silicon chemical shifts with literature data [23] (Figure 4). The HPC product comprised several types of structural units: fully branched T3 units [PhSiO_1.5_], linear T2 units bearing one methoxy or hydroxyl group [PhSiO(OMe)] or [PhSiO(OH)], and terminal T1 units with two functional groups, namely (PhSiO_0.5_(OH)_2_), (PhSiO_0.5_(OH)(OMe)), and (PhSiO_0.5_(OMe)_2_).

The effect of polycondensation conditions on the molecular weight characteristics of the products was evaluated by GPC relative to linear polystyrene standards. The results are summarized in Table 1.

The combined analytical data demonstrate that the reaction of PhTMS under ultrasonic irradiation proceeds via a hydrolytic polycondensation mechanism, as shown in Figure 5, yielding highly functional phenylsilsesquioxane oligomers that are completely soluble in toluene and polar organic solvents such as THF and MTBE.

To confirm the accelerating effect of ultrasound on the HPC of PhTMS, previously demonstrated for MTMS [6], a comparative experiment was performed using ultrasonic irradiation (PhTMS/H_2_O = 1/6, 35 °C, 600 W) and conventional mechanical stirring (PhTMS/H_2_O = 1/6, 35 °C, 500 rpm).

After only 2 h of ultrasonic treatment, the initially heterogeneous reaction mixture became homogeneous, indicating hydrolysis of the alkoxy groups and formation of methanol in amounts sufficient to dissolve all components of the reaction mixture. At this stage, monomer conversion reached 92%, while methoxy group conversion was 79% (sample 16). In contrast, the reaction mixture remained heterogeneous after 72 h of mechanical stirring, and monomer conversion did not exceed 10%. These results are consistent with those previously obtained for MTMS [5]. However, because PhTMS is less reactive than MTMS (a consequence of the steric effect of the phenyl substituent), the irradiation time required to reach the same monomer conversion was substantially longer, from 5 min for MTMS to 2.5 h for PhTMS (sample 17). This lower reactivity also makes HPC more sensitive to process parameters, particularly temperature and ultrasonic power, which affect both monomer conversion and product structure.

Investigation of the effect of ultrasonic power in the range of 300–1200 W on PhTMS HPC and product composition showed that, regardless of the power, complete monomer conversion and high methoxy group conversion (about 90%) were observed at the studied monomer/water ratio (1/6) and temperature (35 °C) (Figure 6A; samples 1–4).

In contrast, the yield of the oligomeric fraction exhibited a non-monotonic dependence on ultrasonic power, with a maximum at 600 W (Figure 6B; samples 1–4). Under these conditions, oligophenylsilsesquioxanes were obtained quantitatively (sample 2). At 300 W, their yield decreased to 77% because of an increased fraction of volatile partial-hydrolysis products of PhTMS and its dimers, indicating insufficient intensification of the polycondensation processes (sample 1). Increasing the power to 1200 W further decreased the oligophenylsilsesquioxane yield to 49%. This decrease was accompanied by an increase in the volatile fraction to 34% and the formation of a precipitate identified as phenylsilanetriol, with a yield of 17% (sample 4). Identification data for the precipitate are provided in the Supplementary Materials (Figures S2 and S3 and Table S2). Apparently, increasing the ultrasonic power affects hydrolysis and condensation differently, favoring hydrolysis of the methoxy groups. This leads to formation of fully hydrolyzed monomer and stabilization of predominantly hydroxyl-containing oligomeric products.

Thus, ultrasonic power is a key parameter in HPC of PhTMS, as in that of MTMS [6]. A power of 600 W provides a quantitative yield of nonvolatile oligophenylsilsesquioxanes, whereas both lower and higher powers decrease the yield.

Temperature was found to be a decisive parameter for intensifying HPC of PhTMS under ultrasonic irradiation. This behavior differs from that of MTMS, for which activation occurs already at 15 °C, providing complete monomer conversion and 95% methoxy group conversion [6]. For PhTMS, ultrasound alone was insufficient at low temperature (Figure 7A): no hydrolysis was detected at 15 °C, monomer conversion reached 60% at 20 °C, and complete monomer conversion, together with 88% methoxy group conversion, was achieved only at 25 °C. Further increasing the temperature to 45–55 °C resulted in a decrease in monomer and methoxy group conversion, reaching 78% and 72%, respectively, at 55 °C (sample 9, Figure 7A).

Under ultrasonic HPC conditions, temperature affects both the chemical and physical (cavitation) processes simultaneously. Increasing temperature initially accelerates the chemical reactions, resulting in increased polycondensation and, consequently, higher molecular weights (samples 2 and 5–9; Figure S4, Supplementary Materials). However, approaching the boiling point of the most volatile component, in this case methanol (bp = 64.7 °C), significantly weakens ultrasonic cavitation because the cavitation bubbles become saturated with vapor and therefore collapse more rapidly, reducing the shock-wave effect [24]. This counteracts the effect of ultrasound as the temperature is increased to 55 °C.

Thus, the temperature study indicates that ultrasound provides effective activation of HPC of PhTMS only within a specific temperature range. Efficient HPC was observed at 25–45 °C. As with power, the effect of temperature on the yield of oligophenylsilsesquioxanes is characterized by an optimum value (35 °C), at which a quantitative yield of oligomeric products is achieved. To obtain the greatest extent of chemical conversion, an optimal balance between temperature and ultrasonic power is required: 35 °C and 600 W (sample 2).

The effect of the initial PhTMS/H_2_O molar ratio (1/3–1/9; samples 2 and 10–13) was then investigated at the optimal ultrasonic power and temperature (600 W, 35 °C, 3 h). Complete monomer conversion was achieved at a minimum PhTMS/H_2_O ratio of 1/4.5 (sample 11), whereas MTMS requires less water (1/3) under comparable conditions [6]. A quantitative yield of oligophenylsilsesquioxanes was observed only at a PhTMS/H_2_O ratio of 1/6 (sample 2) (Figure 8A).

Further dilution of the reaction mixture with water decreased the yield of the oligomeric fraction, as expected (samples 12 and 13; Figure 8B). Nevertheless, oligomers with similar molecular weight characteristics (700–1000 Da) were obtained (Figure S5, Supplementary Materials), with relatively high residual hydroxyl group contents (16–20 wt%) and low methoxy group contents (2–5 wt%) (Figure 8B).

Figure 9A shows the dependence of monomer and methoxy group conversion on irradiation time under the optimal conditions for quantitative formation of oligophenylsilsesquioxanes (PhTMS/H_2_O = 1/6, 35 °C, 600 W; 60–180 min). The data show a gradual increase in monomer and methoxy group conversion with time, accompanied by a gradual decrease in the fraction of partial hydrolysis products (Figure 9B), from 90% at 60 min to complete disappearance by 150 min. At the same time, phenylsilanetriol began to precipitate after 90 min, with its yield gradually increasing to 10% at 120–150 min and disappearing by 180 min. As a result, quantitative formation of oligophenylsilsesquioxane was observed, containing 19 wt% hydroxyl groups and 4 wt% methoxy groups (sample 2).

Thus, a study of PhTMS HPC under ultrasonic irradiation identified 35–45 °C and 600 W as the optimal conditions for the accelerating effect of ultrasound. The optimal PhTMS/water ratio and treatment time providing the maximum yield of oligophenylsilsesquioxanes are 1/6 and 3 h, respectively. Variation in the reaction conditions provides a means of tuning the molecular weight characteristics of the products, primarily by varying the temperature, with relative molecular weights ranging from 600 to 1000 Da (Figure S6, Supplementary Materials). Under the optimum conditions, predominantly hydroxyl-functional oligomers were obtained, containing 17–20 wt% OH groups and 2–6 wt% methoxysilyl groups. Under non-optimal conditions, the methoxy group content increased to 13–40%, while the hydroxyl group content decreased to 1–11 wt%.

Aqueous–alcoholic solutions of oligophenylsilsesquioxanes prepared by ultrasound-assisted HPC of PhTMS did not gel after 5 years of storage. However, the residual functional-group content changed significantly during storage, indicating that polycondensation continued over time (Table 2). As an example, Table 2 summarizes the methoxy and hydroxyl group contents, calculated from elemental analysis data, for sample 8 after 2 and 5 years of storage at room temperature. The aged products remained soluble in toluene, THF, and MTBE.

Thermal treatment of the products obtained by ultrasound-assisted HPC of PhTMS led to rapid curing and formation of an insoluble, glass-like material. Elemental analysis indicated essentially complete consumption of the residual functional groups. For example, the reaction mixture obtained under the conditions used for sample 2 gelled within 1 min at 200 °C.

The thermal stability of sample 8 after 5 years of storage and sample 2 after thermal curing was evaluated by TGA under nitrogen at a heating rate of 10 °C/min. The results were consistent with the assigned chemical structures of the oligophenylsilsesquioxanes (Figure 10). Both samples exhibited high thermal stability characteristic of phenylsilsesquioxanes, with T_5_% values of 477 °C for sample 19 and 525 °C for sample 20. The total mass loss of the room-temperature-aged product (sample 19, Table 2) was 4%, whereas that of the cured product (sample 20) was less than 1% in the temperature range from 30 to 400 °C.

Overall, ultrasound-assisted HPC of PhTMS enables the formation of oligophenylsilsesquioxanes with controlled composition while avoiding volatile partial-hydrolysis products under optimized conditions. Depending on the synthesis parameters, product yields of 60–100% and relative molecular weights of 600–1000 Da were obtained, with methoxy and hydroxyl group contents of 2–6 wt% and 16–20 wt%, respectively. The resulting multifunctional phenylsilsesquioxane oligomers are promising precursors for the preparation of heat-resistant polymer composites.

## 4. Conclusions

This work presents a systematic investigation of the hydrolytic polycondensation of phenyltrimethoxysilane under ultrasonic irradiation in the absence of catalysts and homogenizing solvents at the initial stage of the process. As with MTMS [6], ultrasonic irradiation substantially accelerates hydrolysis and polycondensation of PhTMS compared with conventional mechanical stirring. However, unlike MTMS, accelerating the HPC of PhTMS by ultrasound requires an optimal combination of temperature and power. The key parameters determining the direction and extent of the process were established: irradiation power, reaction-medium temperature, monomer/water molar ratio, and irradiation time. The optimal conditions (PhTMS/H_2_O = 1/6, 35 °C, 600 W, 180 min) were determined, allowing complete monomer conversion and quantitative formation of oligophenylsilsesquioxanes.

Varying the synthesis conditions provides broad opportunities for controlling the composition and molecular weight characteristics of the products. The resulting highly functionalized oligophenylsilsesquioxanes are soluble in organic solvents, have relative molecular masses of 600–1000 Da, and contain controlled amounts of reactive hydroxyl (16–20 wt%) and methoxy (2–6 wt%) groups. The products remain soluble and resistant to gelation during long-term storage, while their residual functional group composition continues to evolve. The oligomers also exhibit high thermal stability, up to approximately 500 °C, supporting their potential use as precursors for heat-resistant binders, protective coatings, varnishes, and enamels.

## Figures and Tables

**Figure 1:**
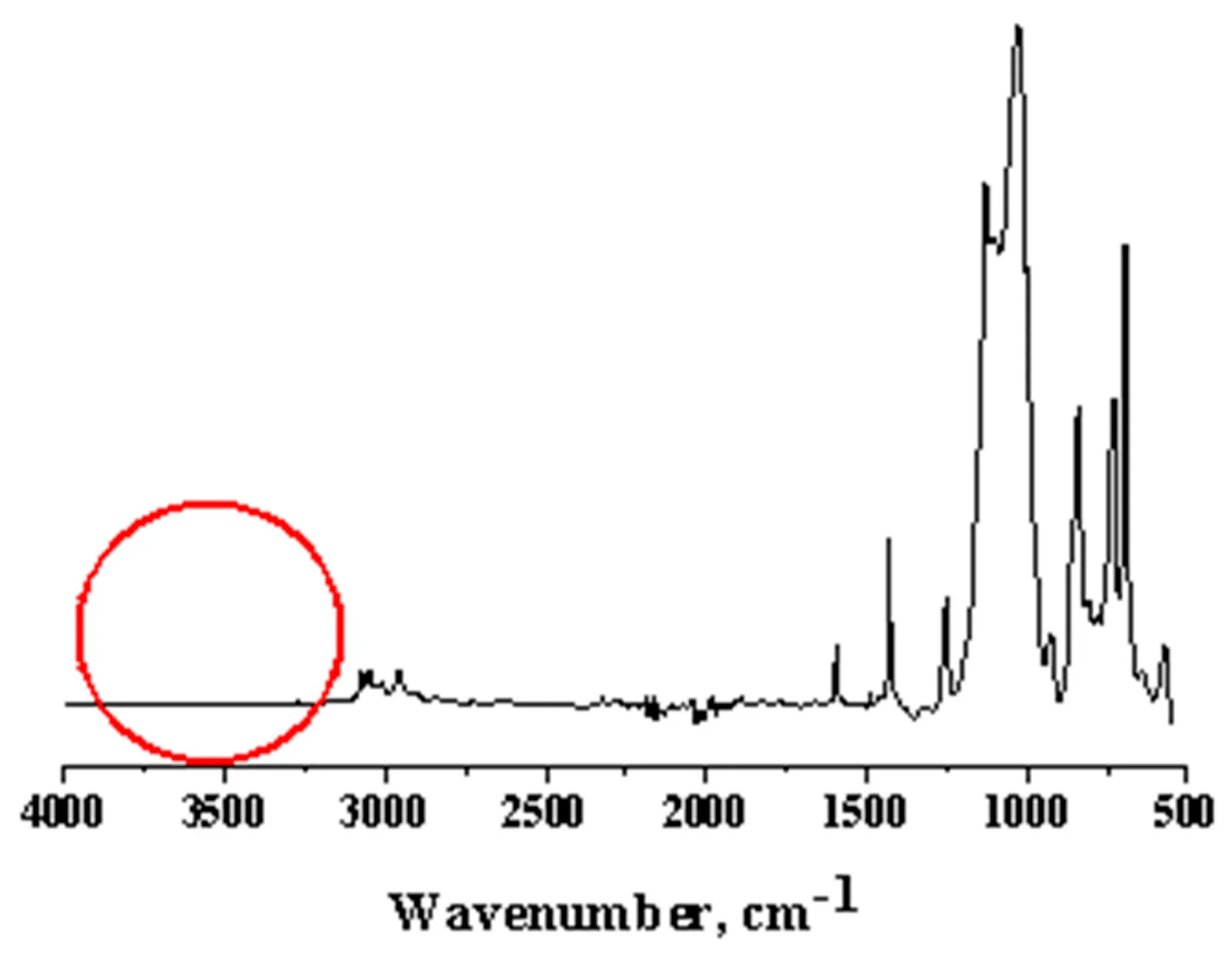
FT−IR spectrum of blocked sample 10.

**Figure 2:**
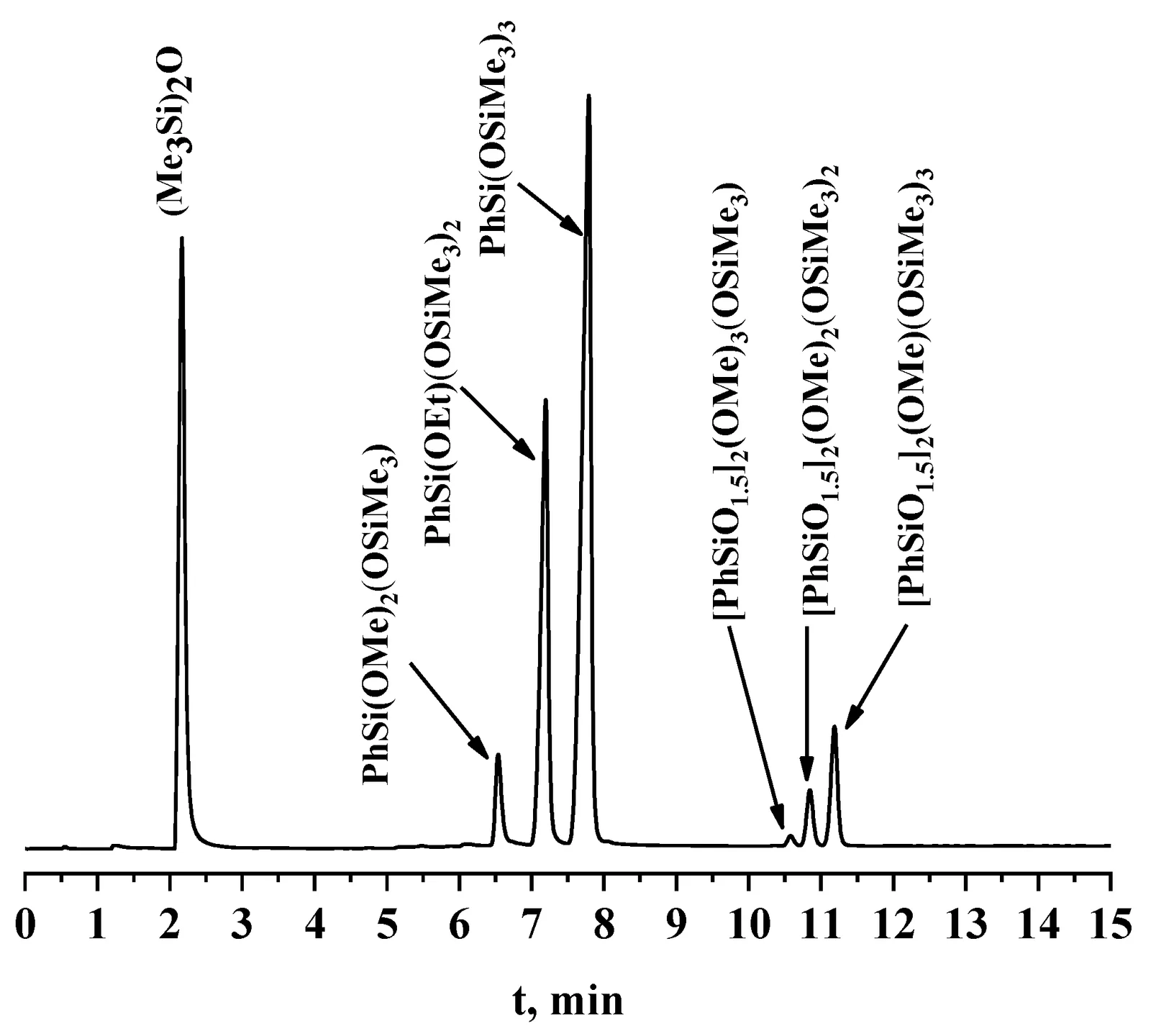
GLC chromatogram of the volatile fraction of sample 10. Peak assignment to specific condensation products was performed by GC−MS.

**Figure 3:**
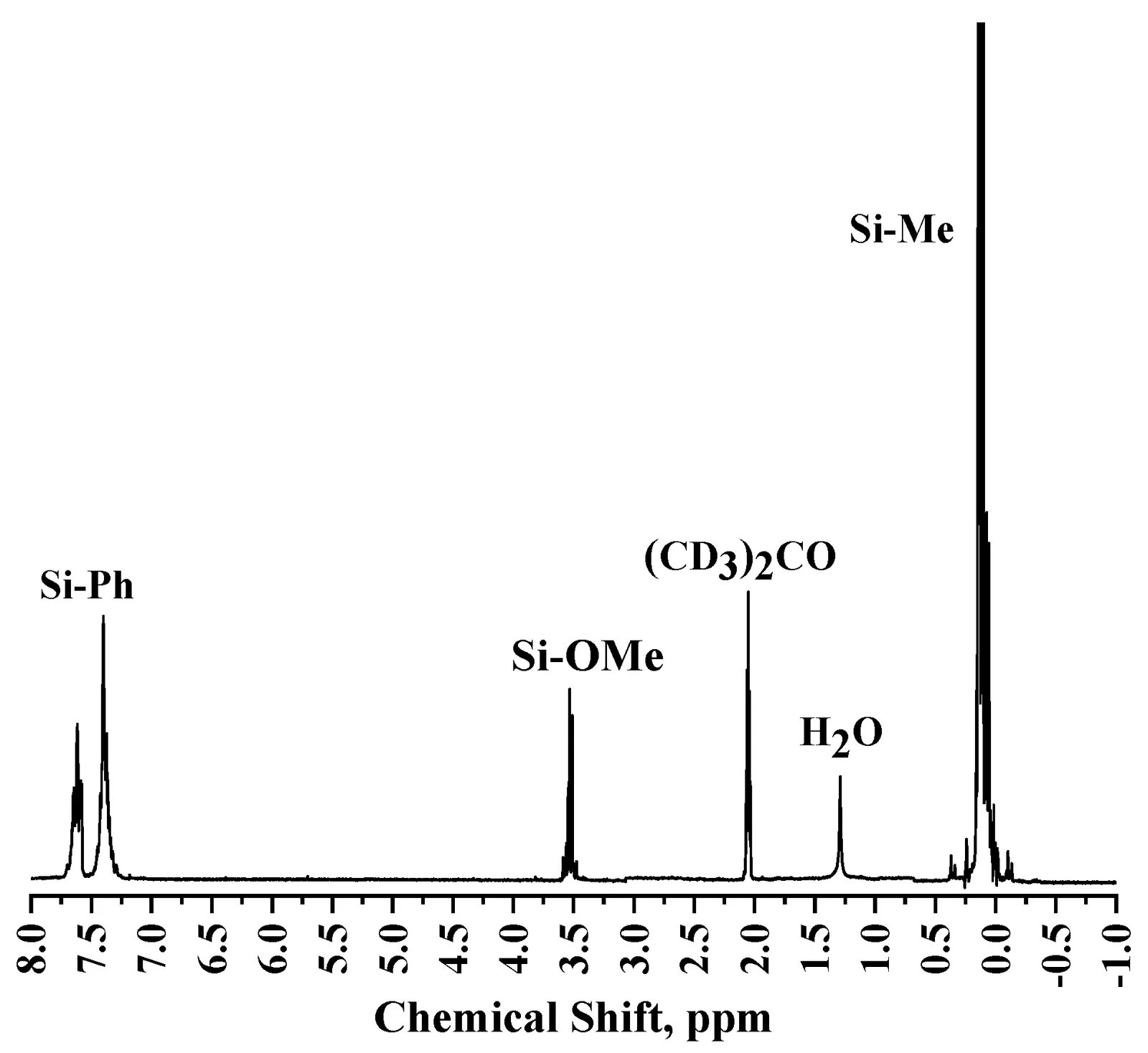
^1^H NMR spectrum of blocked sample 10.

**Figure 4:**
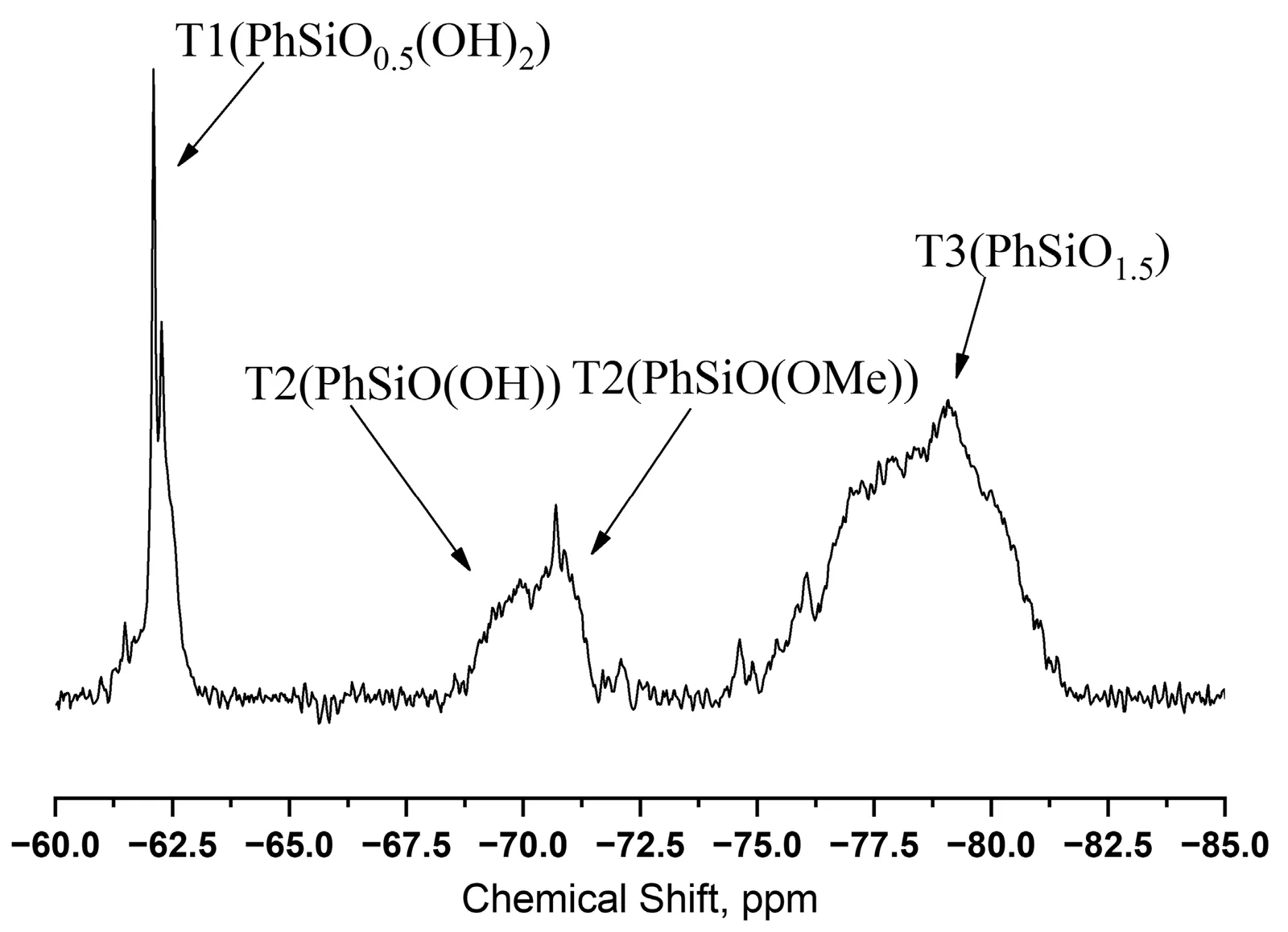
^29^Si NMR spectrum of non−blocked sample 10.

**Figure 5:**
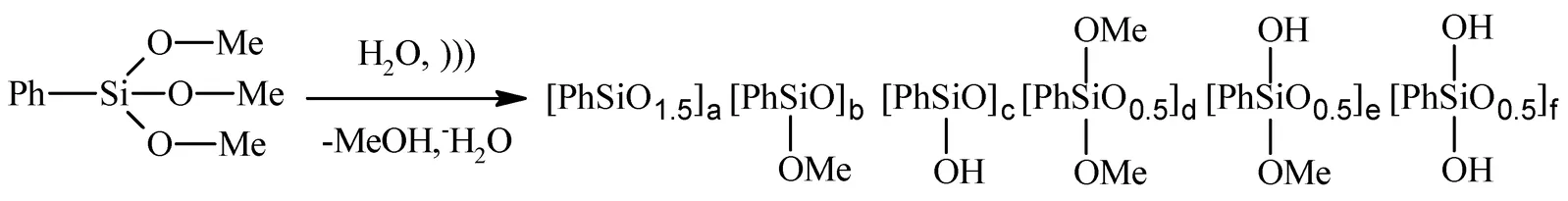
General scheme of catalyst-free hydrolytic polycondensation of PhTMS.

**Figure 6:**
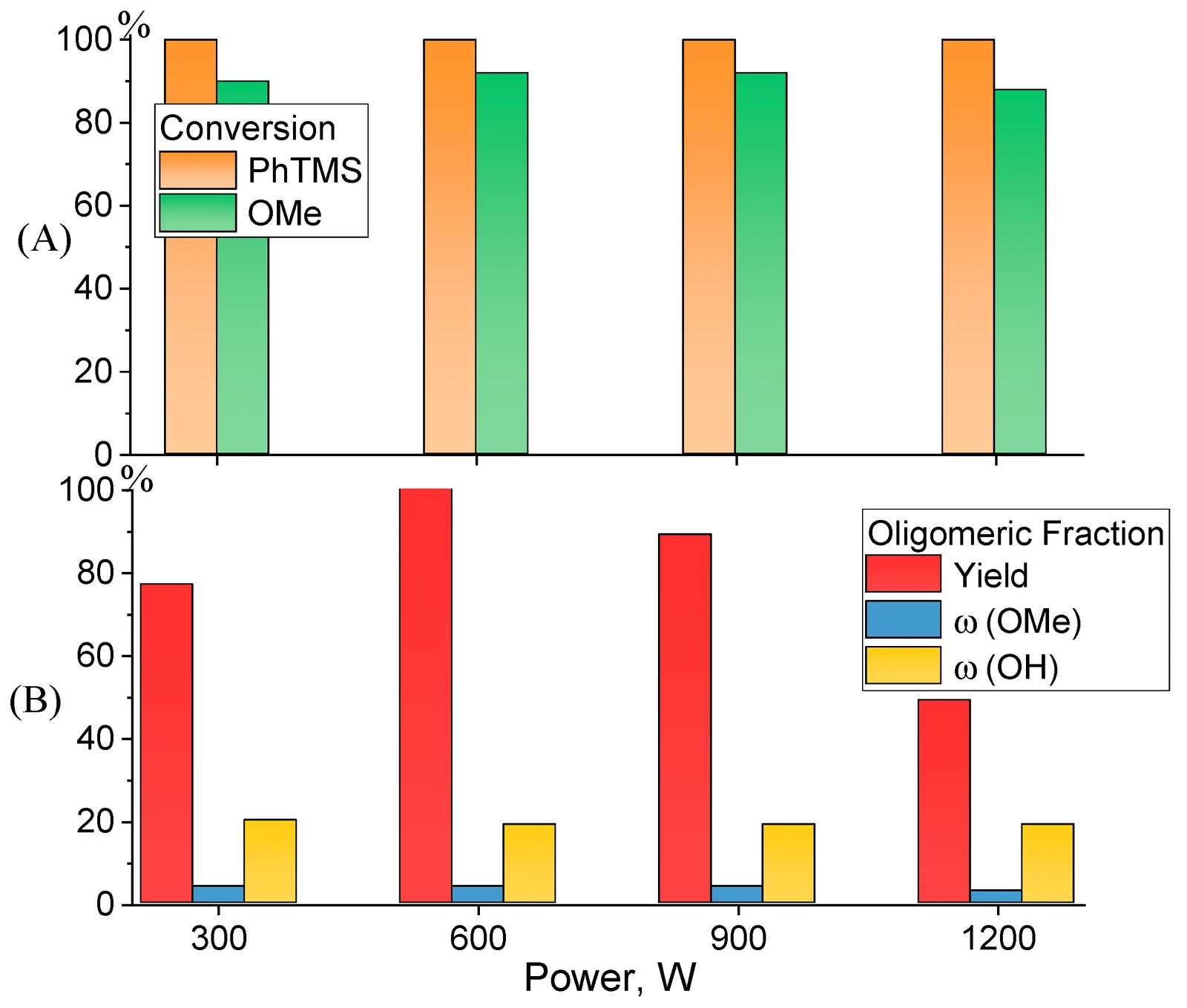
Monomer and methoxy group conversion (**A**), yield and MeO/OH−group content of the oligomeric fraction (**B**), obtained at different ultrasonic power levels (PhTMS/H_2_O = 1/6, 35 °C, 180 min; samples 1–4).

**Figure 7:**
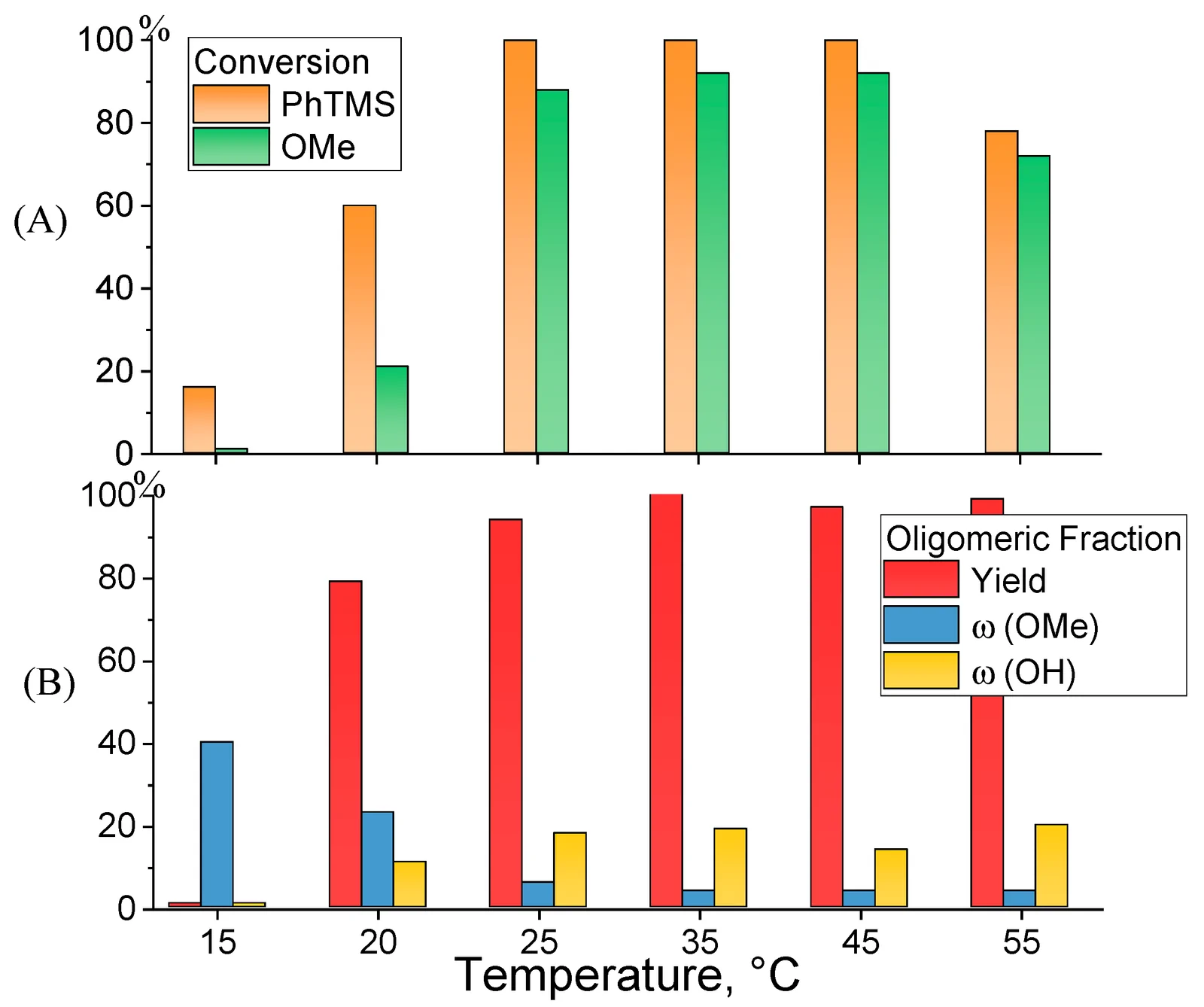
Monomer and methoxy group conversion (**A**), and yield and MeO/OH−group content of the oligomeric fraction (**B**), obtained at different temperatures (PhTMS/H_2_O = 1/6, 600 W, 180 min; samples 2 and 5–9).

**Figure 8:**
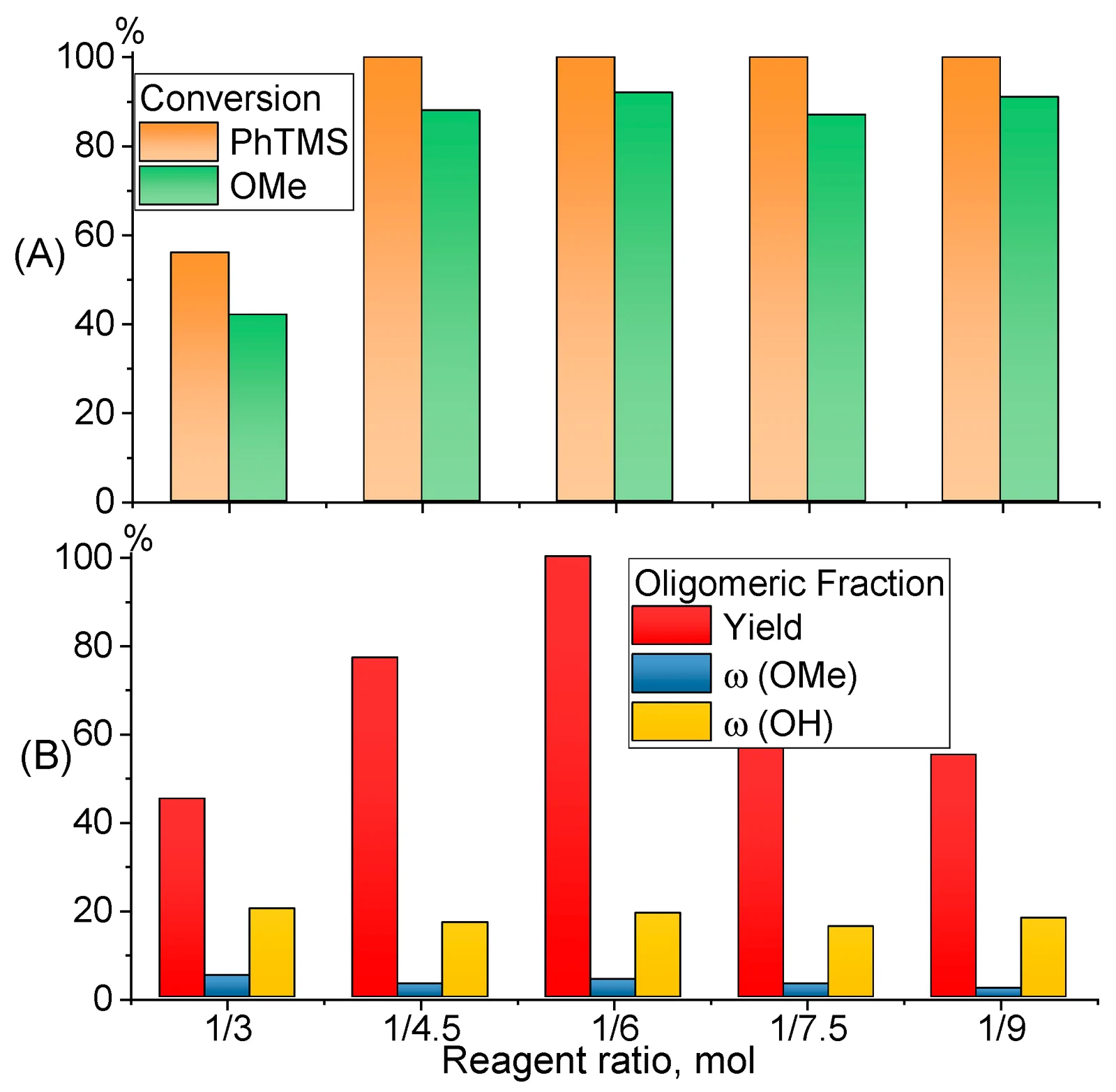
Monomer and methoxy group conversion (**A**), and yield and MeO/OH−group content of the oligomeric fraction (**B**), obtained at different PhTMS/H_2_O ratios (35 °C, 600 W, 180 min; samples 2 and 10–13).

**Figure 9:**
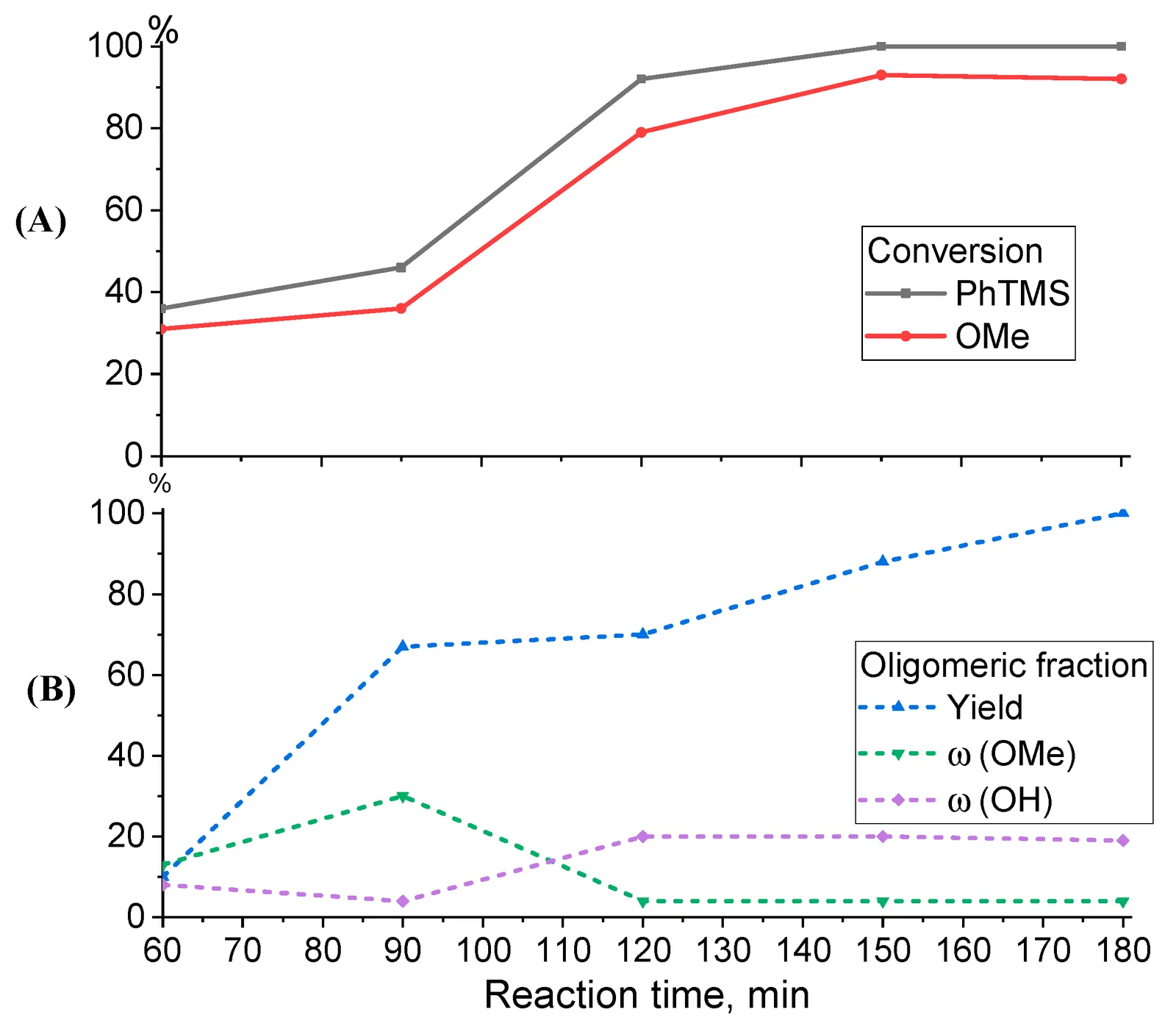
Monomer and methoxy group conversion (**A**), and yield and MeO/OH−group content of the oligomeric fraction (**B**), obtained at different ultrasonic treatment times (PhTMS/H_2_O = 1/6, 600 W, 35 °C; samples 2 and 14–17).

**Figure 10:**
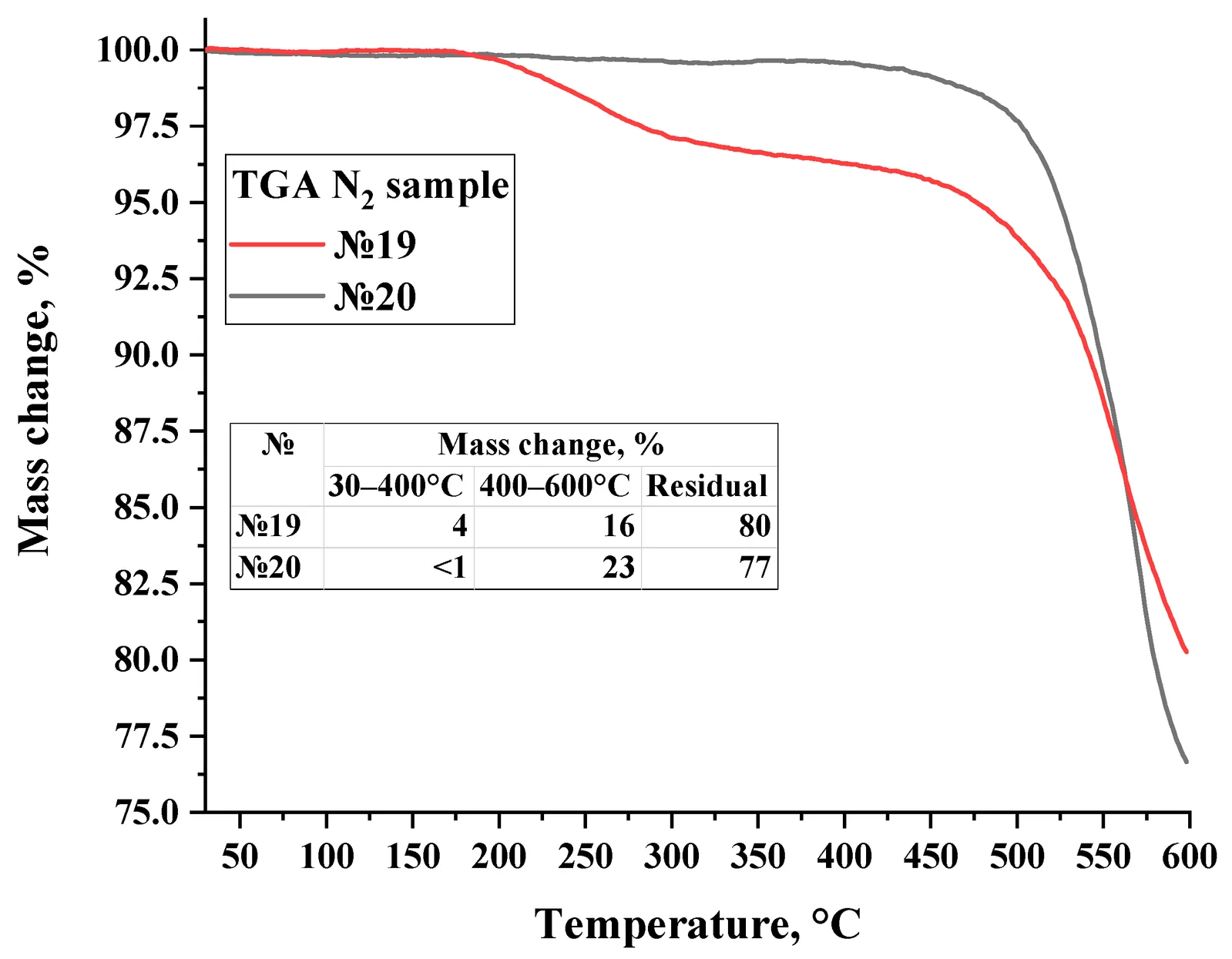
TGA curves of samples 19 and 20 (N_2_, 30−600 °C, 10 °C/min).

**Table 1:** Reaction conditions and characteristics of the products of catalyst-free hydrolytic polycondensation of PhTMS under ultrasonic irradiation.

Sample	Reaction Conditions				Products Characteristics								
	Reagent Ratio PhTMS/H_2_O, mol/mol	T, °C	Power, W	t, min	PhTMS Conversion, %	OMe Groups Conversion, %	Volatile Fraction	Oligomeric Fraction					Solid Fraction *
							Yield **, %	Yield **, %	Mp (GPC)	[MeSiO_1.5_]/ [OMe]/[OH] (^1^H NMR Data)	Content of Groups (wt%)		Yield **, %
											OMe	OH	
Effect of ultrasonic power													
1	1/6	35	300	180	100	90	23	77	900	1/0.22/1.95	4	20	0
2	1/6	35	600	180	100	92	0	100	900	1/0.23/1.83	4	19	0
3	1/6	35	900	180	100	92	1	89	800	1/0.23/1.92	4	19	10
4	1/6	35	1200	180	100	88	34	49	900	1/0.18/1.86	3	19	17
Effect of temperature													
5	1/6	15	600	180	16	1	97	1	600	1/2.80/0.18	40	1	2
6	1/6	20	600	180	60	21	9	79	800	1/1.47/1.21	23	11	12
7	1/6	25	600	180	100	88	3	94	800	1/0.35/1.84	6	18	3
2	1/6	35	600	180	100	92	0	100	900	1/0.23/1.83	4	19	0
8	1/6	45	600	180	100	92	3	97	900	1/0.23/1.67	4	17	0
9	1/6	55	600	180	78	72	1	99	1000	1/0.22/1.27	4	14	0
Effect of reagent ratio													
10	1/3	35	600	180	56	42	55	45	700	1/0.25/1.96	5	20	0
11	1/4.5	35	600	180	100	88	23	77	1000	1/0.17/1.64	3	17	0
2	1/6	35	600	180	100	92	0	100	900	1/0.23/1.83	4	19	0
12	1/7.5	35	600	180	100	87	41	59	1000	1/0.13/1.53	3	16	0
13	1/9	35	600	180	100	91	45	55	1000	1/0.08/1.7	2	18	0
Effect of reaction time													
14	1/6	35	600	60	36	31	90	10	800	1/0.70/0.78	13	8	0
15	1/6	35	600	90	46	36	28	67	1000	1/1.93/0.51	30	4	5
16	1/6	35	600	120	92	79	20	70	700	1/0.23/2.00	4	20	10
17	1/6	35	600	150	100	93	1	88	700	1/0.22/2.02	4	20	11
2	1/6	35	600	180	100	92	0	100	900	1/0.23/1.83	4	19	0

*—In all cases, the solid fraction is phenylsilanetriol. **—the yield (mass content) of the volatile, oligomeric, and solid fractions was calculated as the ratio of the mass of a specific fraction to the total mass of all fractions.

**Table 2:** Synthesis conditions and characteristics of oligophenylsilsesquioxane products.

№	Conditions	Unit Ratio	Group Content (wt%)	
		PhSiO_1.5_/OMe/OH Data ^*^	OMe	OH
8	The product of catalyst-free hydrolytic polycondensation, sample 8	1/0.23/1.67	4	17
18	The product of catalyst-free hydrolytic polycondensation, sample 8, in 2 years at RT.	1/0.05/0.30 *	1	4
19	The product of catalyst-free hydrolytic polycondensation, sample 8, in 5 years at RT.	1/0/0.27 *	0	6
2	The product of catalyst-free hydrolytic polycondensation, sample 2	1/0.23/1.83	4	19
20	The product of catalyst-free hydrolytic polycondensation, sample 2, after curing at 200 °C	1/0/0 *	0	0

*—Data calculated from elemental analysis results.

## Data Availability

The original contributions presented in this study are included in the article and Supplementary Material. Further inquiries can be directed to the corresponding author.

## Supplementary Materials

The following supporting information can be downloaded at https://www.mdpi.com/article/10.3390/polym18192347/s1: Table S1—Reagent loadings, Figure S1— A scheme of the working part of the ultrasonic setup., Figure S2—^29^Si NMR (in DMSO) and IR Spectral Data for the Solid Fraction of Phenylsilanetriol (Sample 8), Figure S3—Thermogram of the solid fraction, Table S2—Elemental Analysis Data for the Solid Fraction. Figure S4— GPC curves of the oligomeric fraction obtained from the HPC of PhTMS at different temperatures PhTMS/H_2_O = 1/6, 600 W, 180 min (№2, 5-9 Table 1) Figure S5— GPC curves of the oligomeric fraction obtained from the HPC of PhTMS at different reagent ratios. Reaction conditions: 35 °C, 600 W, 180 min (№2, 10–13 Table 1). Figure S6— GPC curves of the oligomeric fraction obtained from the HPC of PhTMS at various reaction times. Reaction conditions: PhTMS/H_2_O = 1/6, 35 °C, 600 W (№2, 14–17 Table 1).

## Abbreviations

The following abbreviations are used in this manuscript:

HPC	Hydrolytic polycondensation
PhTMS	Phenyltrimethoxysilane
NMR	Nuclear magnetic resonance
IR	Infrared
GPC	Gel-permeation chromatography
GLC	Gas–Liquid Chromatography
GC-MS	Gas chromatography–mass spectrometry
TGA	Thermogravimetric analysis
THF	Tetrahydrofuran
TMCS	Trimethylchlorosilane
HMDS	Hexamethyldisilazane
FT-IR	Fourier transform infrared
MTMS	Methyltrimethoxysilane
MTBE	Methyl tert-butyl ether
