# Supplementary material for: Catalyst-Free Hydrolytic Polycondensation of Phenyltrimethoxysilane Under Ultrasonic Irradiation
Source: Polymers (Basel). 2026 Sep 26;18(19):2347. doi: 10.3390/polym18192347 (PMC13666611; doi:10.3390/polym18192347)
Supplement: Supplementary file 1 [file polymers-18-02347-s001.zip › polymers-4585295-supplementary.pdf]

Supplementary materials

Table S1. Reagent loadings

№ exp.	PhTMS/H_2O	Reagent loadings, g (mol)	
		PhTMS	H_2O
1	1/6	15,86 (0,08)	8, 64 (0,48)
2	1/6	15,86 (0,08)	8, 64 (0,48)
3	1/6	15,86 (0,08)	8, 64 (0,48)
4	1/6	15,86 (0,08)	8, 64 (0,48)
5	1/6	15,86 (0,08)	8, 64 (0,48)
6	1/6	15,86 (0,08)	8, 64 (0,48)
7	1/6	15,86 (0,08)	8, 64 (0,48)
8	1/6	15,86 (0,08)	8, 64 (0,48)
9	1/6	15,86 (0,08)	8, 64 (0,48)
10	1/3	19,83 (0,10)	5,41 (0,30)
11	1/4,5	19,83 (0,10)	8,11 (0,45)
12	1/7,5	15,86 (0,08)	10,81 (0,60)
13	1/9	13,88 (0,07)	11,35 (0,63)
14	1/6	15,86 (0,08)	8, 64 (0,48)
15	1/6	15,86 (0,08)	8, 64 (0,48)
16	1/6	15,86 (0,08)	8, 64 (0,48)
17	1/6	15,86 (0,08)	8, 64 (0,48)

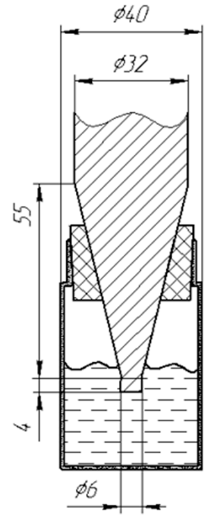

Figure S1 – A scheme of the working part of the ultrasonic setup

Analytical Results for Phenylsilanetriol (Sample 8)

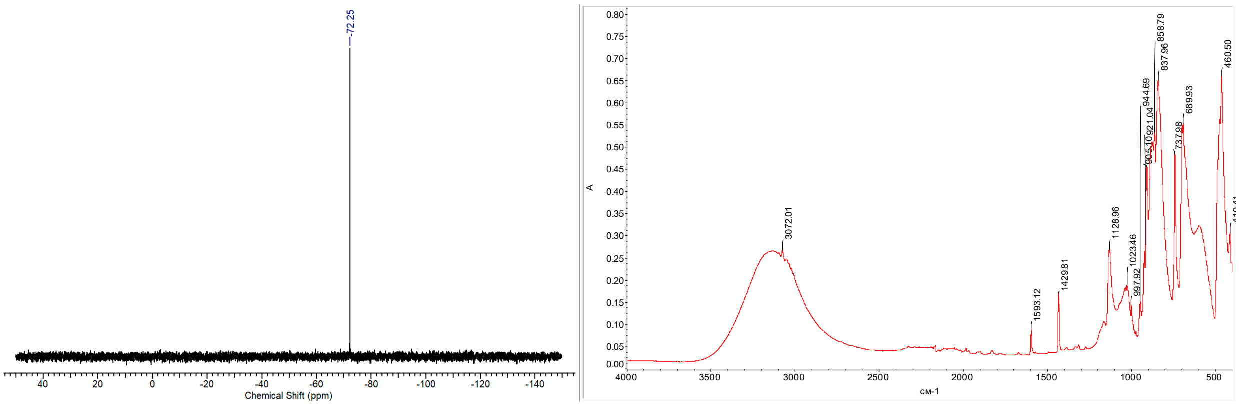

Figure S2 – ^{29}Si NMR (in DMSO) and IR Spectral Data for the Solid Fraction of Phenylsilanetriol (Sample 8)

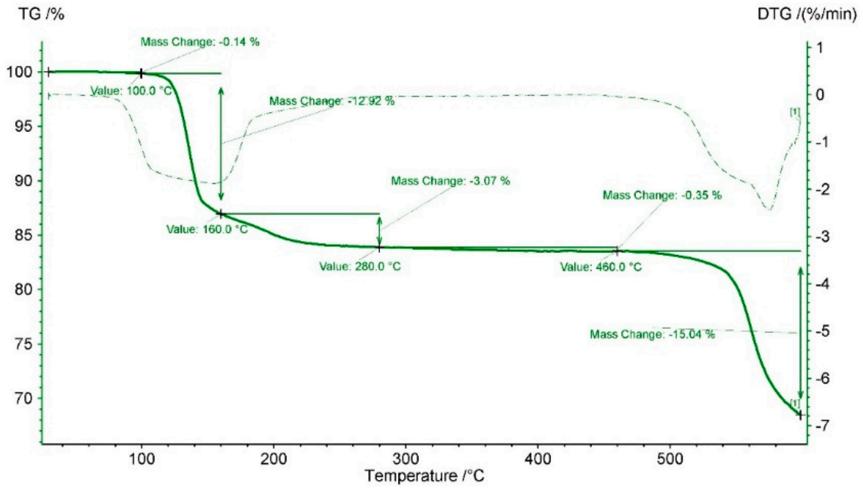

Figure S3 – Thermogram of the solid fraction

Table S2 – Elemental Analysis Data for the Solid Fraction

Element	Value		
	Found	Lit. [70]	Calculated
Si	17.94	17.91	17.98
C	46.36	46.46	46.13
H	5.22	5.64	5.16

GPC curves of the products of the catalyst-free hydrolytic polycondensation of phenyltrimethoxysilane

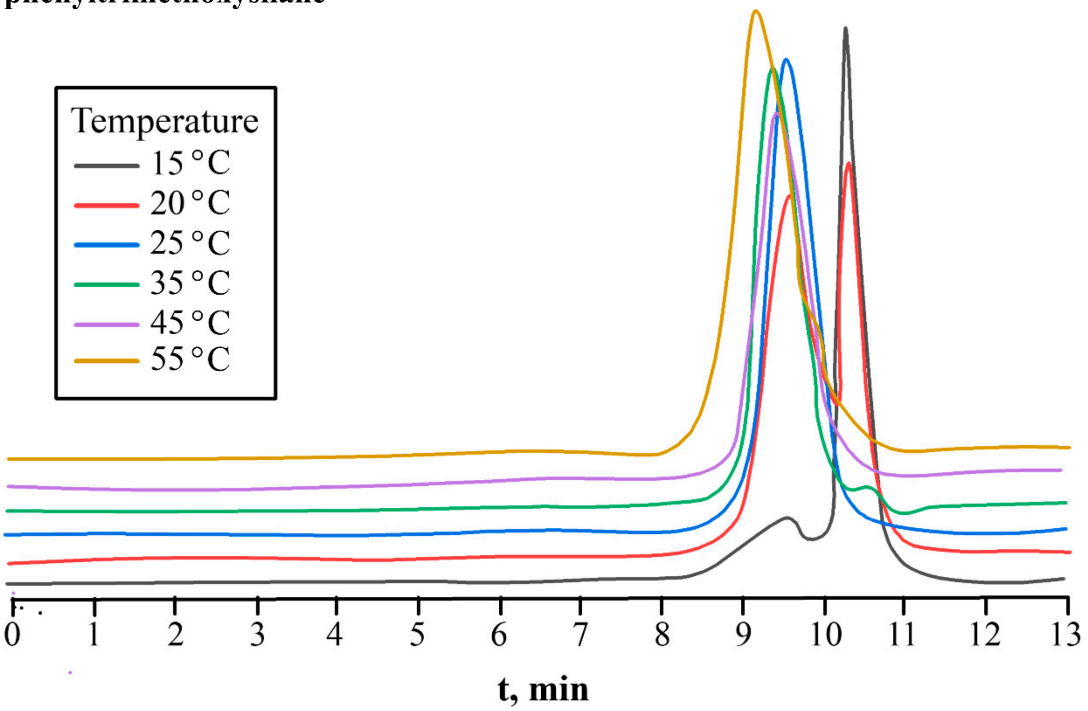

Figure S4. GPC curves of the oligomeric fraction obtained from the HPC of PhTMS at different temperatures PhTMS/H_2O = 1/6, 600 W, 180 min (№2, 5-9 Table 1)

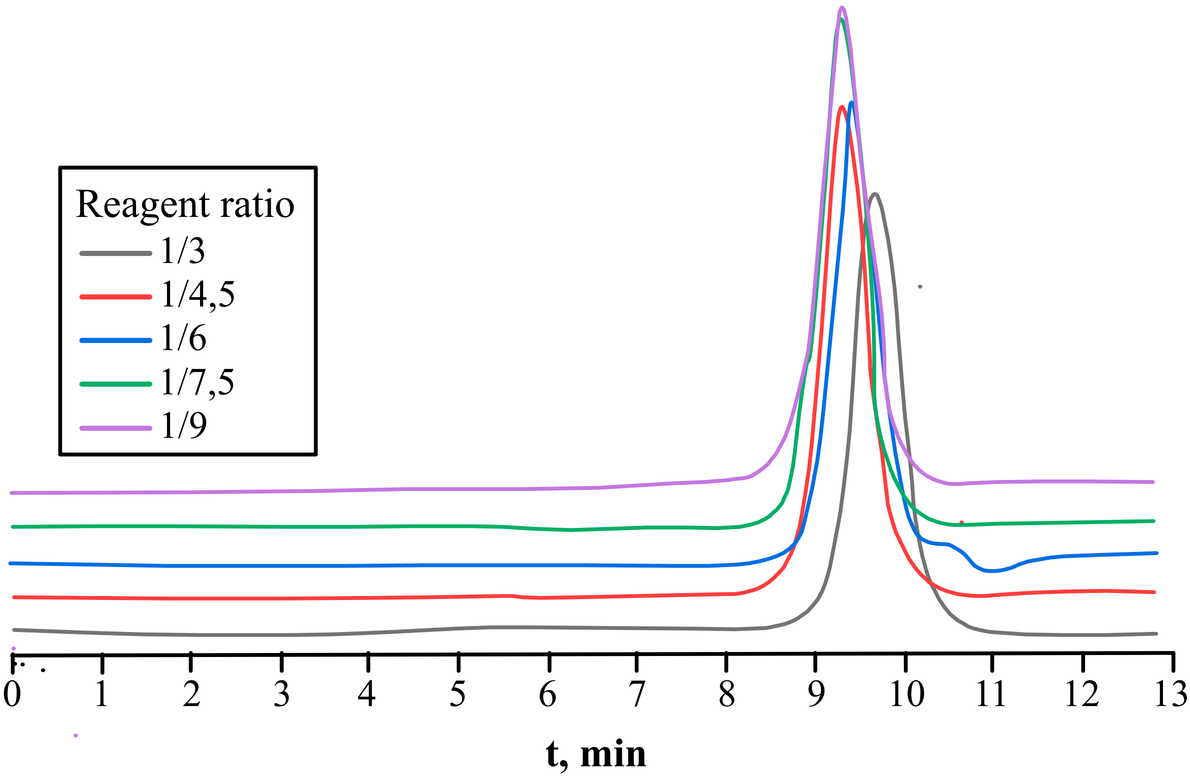

Figure S5. GPC curves of the oligomeric fraction obtained from the HPC of PhTMS at different reagent ratios. Reaction conditions: 35 °C, 600 W, 180 min (№2, 10-13 Table 1).

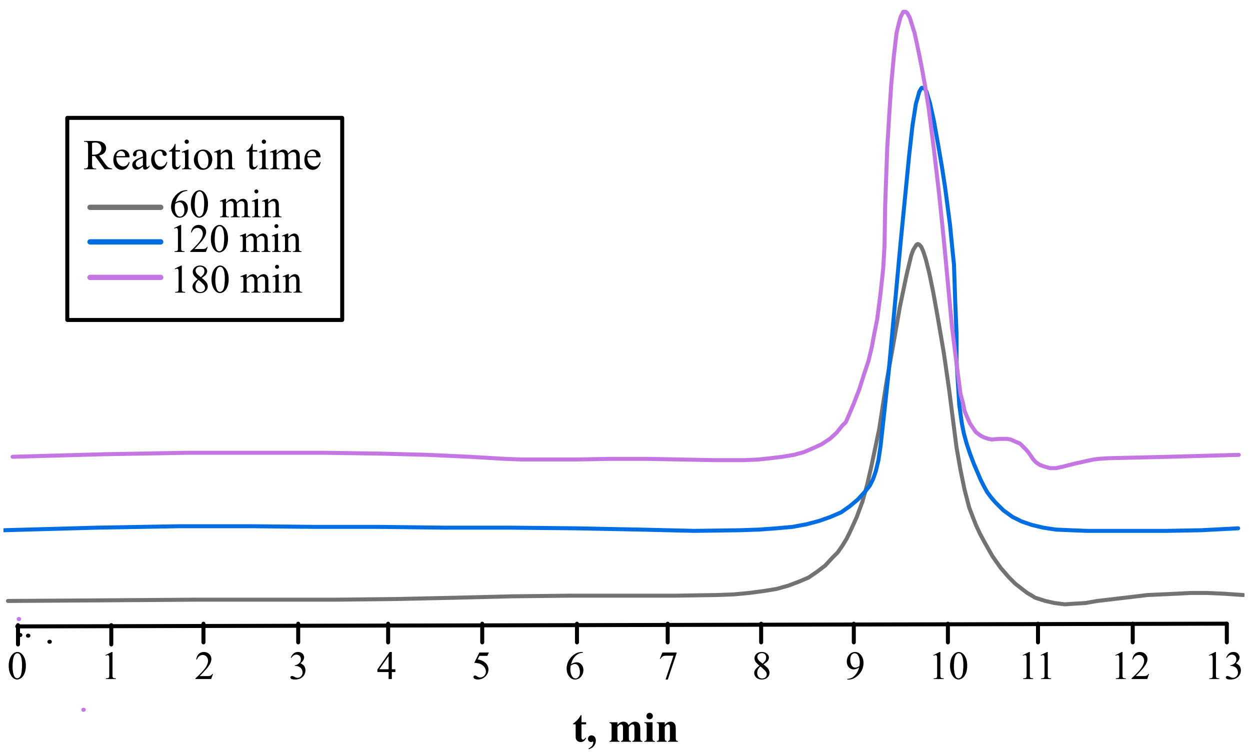

Figure S6. GPC curves of the oligomeric fraction obtained from the HPC of PhTMS at various reaction times. Reaction conditions: PhTMS/H_2O = 1/6, 35 °C, 600 W (№2, 14-17 Table 1).
